# Scrutinizing the Criteria for Character Strengths: Laypersons Assert That Every Strength Is Positively Morally Valued, Even in the Absence of Tangible Outcomes

**DOI:** 10.3389/fpsyg.2020.591028

**Published:** 2020-09-30

**Authors:** Alexander G. Stahlmann, Willibald Ruch

**Affiliations:** Professorship for Personality and Assessment, Department of Psychology, University of Zurich, Zurich, Switzerland

**Keywords:** positive psychology, VIA, experiment, moral judgment, deontology, consequentialism, utilitarianism, process dissociation

## Abstract

This study examines Peterson and Seligman’s (2004, p. 19) claim that every VIA character strength “(…) is morally valued in its own right, even in the absence of obvious beneficial outcomes”. Although this criterion assumes a pivotal role in distinguishing character from personality, no previous study has investigated its validity. Based on what Peterson and Seligman (2004) have provided us with, we describe how we built our study around indirectly testing every strength’s assumed moral evaluation, in which inclinations toward deontology (e.g., “torture is wrong regardless of tangible positive outcomes”) and consequentialism (e.g., “torture can be good if it accounts for more positive than negative outcomes”) may play a critical role. We used Peterson and Seligman’s (2004) handbook to construct four ultra-short stories for every strength: the stories depict various agents engaging in strength-related behavior (e.g., a young student courageously stepping up against school bullies). We prompted participants to rate these and twelve anchor stories multiple times as to whether the agents acted morally correct: In the first block, the actions’ consequences were undetermined while in the second block, the actions had either positive, negative, or mixed consequences, which we used to compute proxies of participants’ inclinations toward deontology and consequentialism. The ratings of *N* = 230 German-speaking laypersons suggest that the criterion stands: participants perceived every strength as positively morally valued when consequences were undetermined, and positive consequences did not account for or increase this effect. However, moral value seems to come in degrees, and some strengths were valued more strongly than others (top five: judgment, honesty, kindness, fairness, and hope). Furthermore, specific character strengths (measured by self-report) were connected with more positive evaluations (e.g., endorsing spirituality was connected with rating spirituality as more positively valued). Both deontology and consequentialism were connected with more positive evaluations, and we suggest two hypotheses to explain how such inclinations can lead to perceiving character strengths as positively valued. Our findings highlight the importance of scrutinizing the criteria for character strengths, and our experimental paradigm can offer a template to further investigate character strengths’ moral evaluation and other fundamental assumptions in upcoming studies.

## Introduction

About 15 years ago, Peterson and Seligman’s (2004) seminal handbook and classification brought about a renaissance of the concept of character–a quality that has long been written off in personality research. Today, the abundance of studies into the positive outcomes of VIA character strengths proves that character matters: Character strengths constitute well-being (e.g., Park et al., 2004a; Wagner et al., 2019), contribute to work performance and academic achievement (e.g., Park and Peterson, 2006; Littman-Ovadia and Lavy, 2016), and build resilience toward life’s hardships, illness, and loss (e.g., Peterson et al., 2006; Martínez-Martí and Ruch, 2017). Indeed, there is also emerging evidence that character strengths can contribute to sustainability and pro-environmental behavior (e.g., Corral-Verdugo et al., 2015; Moeller and Stahlmann, 2019). However, although we know a lot about what character *can do*, we know surprisingly little about what character *is*, and what sets it apart from other individual differences. In fact, nearly all of the positive outcomes above also pertain to the Big Five personality traits: for example, extraversion and neuroticism predict subjective well-being (e.g., Diener and Lucas, 1999), conscientiousness contributes to job performance (e.g., Barrick and Mount, 1991), and all Big Five traits are differentially connected with resilience (Oshio et al., 2018). Research into such positive outcomes reinstalled the concept of character in the literature, but it did not deepen our understanding of what makes character unique.

This gap in our knowledge is irritating because Peterson and Seligman (2004) have provided us with a list of criteria that should define what character is. These criteria emerged in the process of selecting entries for the VIA classification–to consolidate their common factors and distinguish them from seemingly related concepts, such as talent, ability, and personality (see Peterson and Seligman, 2004, pp. 16–28). The literature currently counts twelve of such criteria (e.g., Park, 2018, pp. 4–5)–some of which simply serve to ground character in the framework of individual differences, such as *traitlike* (“is an individual difference with demonstrable generality and stability”), *measurable* (“has been successfully measured by researchers as an individual difference”), and *distinctiveness* (“is not redundant [conceptually or empirically] with other character strengths”). Similar to positive outcomes, these criteria have received considerable scientific scrutiny (e.g., traitlike: Gander et al., 2019; measurable: McGrath, 2016; distinctiveness: McGrath, 2014), presumably because they pertain to all individual differences and there are hence established methods to evaluate them. Other criteria are rather unprecedented and character-specific, such as *morally valued* (“is valued in its own right and not as a means to an end”), *does not diminish others* (“elevates others who witness it, producing admiration, not jealousy”), and *paragons* (“is strikingly embodied in some individuals”). These are arguably the key criteria that make character unique, yet there has been little discussion about their validity and even less research into whether character strengths can indeed satisfy them (Ruch and Stahlmann, 2019).

Superficially, these criteria may seem to be rather obvious and straightforward–it is not hard to come up with several examples of individuals who presumably endorsed certain strengths to a remarkable degree, such as Pablo Picasso (creativity), Viktor Frankl (hope), or Arnold Schwarzenegger (self-regulation). However, the criteria also offer a more hidden, extensive perspective, which becomes apparent when we ask, for example, *why* some people can grow to become such paragons of character, or *how* their actions can inspire so many others around the world to follow in their footsteps. Scrutinizing the criteria shows us that they cannot only *describe* character but provide us with the questions whose answers allow us to *explain* it. In other words: not only the correlations with sensible outcomes, but especially the criteria are key to proving that character matters. Accordingly, we need to rigorously explore these criteria, and hence this account seeks to exemplify how one of them–morally valued–can be investigated in an experimental framework.

### Morally Valued Is One of the Most Defining Yet Understudied Criteria of Character Strengths

Among the key criteria, *morally valued* can be assumed a special role because it reflects a historic paradigm shift in personality research: At the beginning of the 20th century, Gordon Allport^1^ saw himself confronted with a surge of interest into the study of what was then referred to as human nature (see Allport, 1921; Allport and Vernon, 1930). In an attempt to consolidate the diverse literature and connect personality psychology with the methods of natural science, he assumed famous behaviorist Watson’s (1919) perspective that “character is defined (…) as the personality evaluated according to prevailing standards of conduct” and that “psychologists who accept Watson’s view have no right (…) to include character study in the province of psychology.” (Allport, 1921, p. 443). It is important to note that Allport (1921) did not “banish” all such evaluative traits from personality research: “Terms which originated in social judgment (…) may and often do, become ideals or guiding principles adopted by individuals. In this sense the introception of an ethical ideal into subjective attitude turns a characterial designation into a true trait-name” (Allport and Odbert, 1936, p. 28) and hence qualifies it for psychology. However, he did deter from research into whether psychology can and should consider such traits *morally valued* because “(t)he same behavior (…) may be moral in one locality, immoral in another, moral at one period of time, immoral at another” and “(t)here are no ‘moral traits’ until trends in personality are evaluated” (Allport, 1927, p. 285). Peterson and Seligman (2004) acknowledged Allport’s (1921) differentiation according to such standards of conduct, but they rejected his deduction because they purposefully designed the VIA classification to only include entries that pertain to ubiquitously^2^ shared virtues–in contrast to culture-specific and temporarily prevailing standards (Peterson and Seligman, 2004, pp. 33–52). Indeed, they argue that “(t)he ubiquity of these core virtues suggests the possibility of universality and eventually a deep theory about moral excellence phrased in evolutionary terms.” (Peterson and Seligman, 2004, p. 51). In this sense, character strengths’ assumed moral value is rightfully one of the key criteria that distinguish them from other individual differences.

Earlier, we stated that there had been little quantitative analysis of the key criteria, and this is especially the case for *morally valued*: The literature frequently mentions it as a central one, including two consecutive editions of The Oxford Handbook of Positive Psychology (Peterson and Park, 2009; Park, 2018). However, the only attempts at critical evaluation can be found in Peterson and Seligman’s handbook (2004) and, to a lesser extent, in a book contribution by Park and Peterson (2007), in both of which every character strength was theoretically rated according to whether it satisfies this criterion. Surprisingly, an increasing number of strengths were not considered inherently morally valued because they are believed to only “become morally valued when coupled with other strengths in the classification” (Peterson and Park, 2011, p. 52). Such strengths were referred to as *value-added* (e.g., Peterson and Park, 2011, p. 52), and Table 1 summarizes the literature on them. Notably, where Peterson and Seligman (2004) had used careful language, the more recent literature (e.g., Park, 2018) presents the existence of several of such value-added strengths (approx. ¼) as a fact. So far, this striking contradiction to the classifications’ fundamental principles seems to have been tolerated as a kind of peculiarity, but if we were to take the criteria seriously, this would necessitate one of two far-reaching implications: We would have to redefine or altogether abandon either (1) the criterion or (2) the strengths in question. It is hence pivotal to examine whether this criterion applies to all strengths–that “(a)lthough strengths can and do produce desirable outcomes, each strength is morally valued in its own right, even in the absence of obvious beneficial outcomes” (Peterson and Seligman, 2004, p. 19).

**TABLE 1:** List of character strengths that have previously been discussed as value-added, including all references in the literature and excerpts of the reasoning.

**Character strength**	**Literature**	**Excerpt/reasoning**
Perseverance	Peterson and Seligman, 2004; Park and Peterson, 2007	“Persistence is morally valued. We admire the busy bee, the tortoise but not the hare, the little engine that could, and Rocky Balboa answering the bell again and again (…). At the same time, we acknowledge a downside to diligence when it takes the form of perseveration. Aristotle’s doctrine of the mean reminds us that too much diligence can be as much a vice as too little (…).” (Peterson and Seligman, 2004, p. 203)
Zest	Peterson and Seligman, 2004; Park and Peterson, 2007; Peterson and Park, 2009, 2011; Park, 2018	“We hesitate to conclude that enthusiasm *per se* is morally valued; this judgment will usually follow only when the activity pursued with enthusiasm is itself moral. However, if life lived well–with vigor and energy–is a good thing, and of course it is, then perhaps enthusiasm in these terms is morally valued.” (Peterson and Seligman, 2004, p. 210)
Humor	Park et al., 2004a; Park and Peterson, 2007; Peterson and Park, 2009, 2011; Park, 2018	“Strengths such as humor and zest are not morally valued in their own right but become morally valued when coupled with other strengths in the classification. So, a humorous person is simply funny, but a humorous person who is kind is very special and morally praiseworthy. We call these value-added strengths and intend to study them further.” (Peterson and Park, 2011, p. 52)
Humility	Park et al., 2004b	“Perhaps modesty is what we term a value-added strength, not especially satisfying in its own right but–like humor, for example–important when coupled with other well-developed strengths of character. However, we tested this possibility by creating all possible product terms between modesty and the other 23 strengths in our classification and found no evidence that these interactions were associated with life satisfaction beyond the contribution of their components.” (Park et al., 2004b, pp. 631–632)
Love of learning Curiosity Appreciation of beauty	*Park and Peterson, 2007; Park, 2018	“Although character strengths are generally defined as morally valued traits, several character strengths in the VIA Classification are positive traits but not moral traits, such as love of learning, curiosity, and appreciation of beauty.” (Park, 2018, p. 5)

*Love of learning was already marked as potentially value-added by Park and Peterson (2007), although they did not explain this decision.

### Designing a Study to Evaluate Character Strengths’ Assumed Moral Value

As there is no standard procedure for such an examination, we have to work with what Peterson and Seligman (2004) have provided us with and carefully build our study around testing what we believe they deemed to be the criterion’s fundamental qualities. It is clear that they put particular emphasis on character strengths’ moral integrity in the absence of positive consequences. Accordingly, we can assert that a proper study should contrast such scenarios from those in which character strengths *do* produce positive consequences–for example, by experimental manipulation. Other issues leave more room for interpretation and may result in different design options. From a psychological perspective, these issues should primarily pertain to two critical questions: *How* should we measure character strengths’ moral evaluation, and *whom* should we invite to give their rating? In the following paragraphs, we will describe how we resolved these issues and how our reasoning guided our study design.

#### How Should We Measure Character Strengths’ Moral Evaluation?

Ignoring the second question for the moment, the most straightforward way to measure character strengths’ moral evaluation seems to involve asking individuals *directly* whether they believe that traits such as bravery, kindness, and spirituality are morally valued. A similar approach was used, for example, by Biswas-Diener (2006) in a study on whether VIA character strengths are also recognized in less often studied communities, such as the Kenyan Maasai or Inughuit in Northern Greenland. However, although efficient, this approach presumably does not come without a cost to validity and reliability: as every other trait, character strengths refer to dispositions toward a range of discrete emotions, behaviors, cognitions, and desires (see Wilt and Revelle, 2015), and it is not known, but unlikely, that individuals unanimously share the same cognitive representations. This is one of the main reasons why items in personality inventories (including “character inventories,” such as the VIA Inventory of Strengths; Peterson and Seligman, 2004) are anchored in specific contexts and situations: standardizing the frame of reference makes it more likely that participants are considering the same concepts as the researchers have when giving their response. Therefore, we concluded that character strengths’ moral value should be rated *indirectly* by judging agents’ actions in multiple well-defined scenarios instead of providing only one abstract rating per strength. The rating itself should presumably be given using a *bipolar* answer format (i.e., ranging from *immoral* [−] to *amoral* [0] and then to *moral* [+]) to avoid steering participants toward artificially-inflated positive evaluations (e.g., by only allowing participants to rate how *positively* morally valued the agents acted). However, as participants may hence inadvertently feel required to use the full scale, we also suggest including additional scenarios that pertain to immoral or amoral actions. Such scenarios may map onto actions that Peterson and Seligman (2004, p. 299) called “(…) talent(s) or abilit(ies) that fall outside the moral realm” (e.g., general intelligence, athletic ability, or perfect pitch) or even those that are antithetical to the concept of good character (e.g., the Dark Triad traits Machiavellianism, narcissism, and psychopathy; for an overview, see Paulhus and Williams, 2002; Furnham et al., 2013).

#### Whom Should We Invite to Give Their Rating?

In order to resolve the question of whom should judge character strengths’ moral evaluation, we need to discuss why Peterson and Seligman (2004) seem to have been so keen on stressing that character strengths’ moral integrity is untouched by tangible positive consequences (or the lack thereof). Although they did not draw an explicit connection (neither in the handbook nor in later literature), their wording strongly implies that they wanted to make an argument for the principle of *deontology* (see Kant, 1785/2007)–or at least against the principle of *consequentialism* or *utilitarianism* (see Mill, 1861/1998): according to Kantian deontology, an action that produces positive consequences can never be considered moral if it cannot generalize to a universal principle of conduct. For example, torture would never be considered moral, because if everyone would torture to acquire information, then no one could feel safe and torture would have to be reliably expected. In contrast, Millian consequentialism would consider those actions moral that produce more net good than any alternative actions. In the example above, if torturing an alleged terrorist would lead to saving the lives of innocent civilians and there are no alternative actions that yield more net good, torture could be considered moral. However, this is where Peterson and Seligman (2004) put their criterion: By emphasizing that the ends do not sanctify the means but that the moral value lies in the exercise of character strengths themselves, they essentially imply that character strengths are deontological by nature, and that every strength map onto such a universal principle of conduct.

Indeed, a consequentialist perspective would strongly challenge the criterion as it stands now (and by extension also Allport’s differentiation): instead of a characteristic of the strengths, the moral value would be a characteristic of their consequences or the contingency between strengths and consequences. However, this poses a problem because we can assume that individuals who lean toward consequentialism may *reject* character strength’s moral evaluation unless the strengths account for tangible positive consequences. Moreover, such individuals could judge character strengths that accidentally produce negative consequences (irrespective of the actor’s good intentions) as *negatively* morally valued. Even individuals who lean toward deontology may not necessarily judge character strengths as positively morally valued because they may not believe that the strengths can generalize to universal principles of conduct. In this sense, Peterson and Seligman’s (2004) criterion is as much a set of assumptions about character strengths as it is about ethical decision making in general, and thus the question of whom to invite to give their rating quickly becomes a question of defining moral value as a whole–and as several studies have shown that individuals differ in their inclinations toward deontology and consequentialism (see, e.g., Tanner et al., 2008; Conway and Gawronski, 2013), this may be the most important predictor of whether character strengths will pass or fail the criterion.

To make matters even more complicated, the relevant literature has long abandoned the purely rationalist perspective on ethical decision making that was prevalent in the late 20th century (see, e.g., Kohlberg, 1981, 1984). More contemporary models, such as Greene’s dual-process theory (see, e.g., Greene, 2007; Conway and Gawronski, 2013), rather stress the importance of immediate affective reactions, available cognitive resources, and motivation, that may or may not enable rationalist processing. If emotions such as happiness, sadness, and anger (Gawronski et al., 2018) or other factors such as time pressure (Gawronski and Beer, 2017) or even emotion-related damage in the ventromedial prefrontal cortex (Greene, 2007) can influence ethical decision making, it becomes not only a question of *whom* to invite but also of *when* to invite them. This also raises the question whether individual differences in *trait* character strengths–such as the endorsement of bravery or kindness–can influence affective responses and cognitive processing in a similar fashion: for example, a certain degree of perceived similarity between actor and judge may lead to more positive evaluations (“It takes one to know one”).

Altogether, these considerations emphasize that Peterson and Seligman’s (2004) criterion can only be met if a set of specific assumptions about the judges’ processing of strength-related actions can be met as well: specifically, this would only be possible if character strengths’ moral value were not only independent of tangible positive outcomes but also of individual differences in ethical decision making that map equally well onto inclinations toward deontology and consequentialism *and* intuitive, affective heuristics. Ideally, we can hope that our account pushes toward more research that addresses all of these potential factors in dedicated studies–either by actively manipulating or by controlling them. For now, we can only lay the groundwork by investigating whether Peterson and Seligman’s (2004) criterion can still hold–notwithstanding all the factors that seem to challenge its validity. To do so, we decided to unselectively invite individuals whom we recruited from the general population to give their rating–this should include individuals with diverse ethical inclinations and in various affective, cognitive, and motivational states. However, we did decide to additionally measure inclinations toward deontology and consequentialism as these seem to have been so significant for Peterson and Seligman’s (2004) reasoning. We also decided to measure trait character strengths because they constitute the conceptually closest other factors that could constitute differences in moral evaluations.

### Aims of This Study

Based on our considerations above, we took the first step toward systematically investigating character strengths moral value by designing an online experiment that is explicitly aimed at measuring individuals’ moral evaluation of fictional agents’ strength-related behavior. First, we tested whether Peterson and Seligman’s (2004) claim was correct in that every strength is generally recognized as positively morally valued, even in the absence of positive consequences. Second, we tested whether mixed (positive *and* negative) and negative consequences can affect participants’ evaluations and translated individual response patterns into scores that map onto their inclinations toward deontology and consequentialism. Third, we tested whether these inclinations and individual differences in trait character strengths are connected with individuals’ moral evaluations. Following up on our theoretical reasoning, our study provides first evidence of whether the criterion is meaningful, whether it can be met by all strengths, and whether there are other factors that influence character strengths’ moral evaluation.

## Materials and Methods

### Participants and Procedure

This study used a convenience sample comprising *N* = 230 participants (80.00% female, 20.00% male; *M*_age_ = 25.01, *SD*_age_ = 6.25, range = 18–45 years). The majority were Germans (56.52%), Swiss (27.83%), German-speaking Italians from South Tyrol (5.22%), and Austrians (4.34%). About three quarters were college or university students (78.26%) who had received upper secondary education (undergraduate: 74.44%) or tertiary education (graduate: 25.56%). The remainder had largely received tertiary education (Bachelor or higher: 85.71%) or upper secondary education (14.29%).

The experiment was administered online via www.soscisurvey.de in January and February 2020. Participants had to be at least 18 years old and fluent in German. They provided informed consent before participation, began by providing demographic data, and subsequently worked on the VIA-IS and the CS-MET. They were debriefed after participation and received partial course credit upon request. The experiment took participants approximately 1 h to complete.

### Measures

#### Character Strengths’ Moral Evaluation

We created the Character Strengths’ Moral Evaluation Task (CS-MET) to assess the VIA character strengths’ moral evaluation. The CS-MET’s basic building blocks are 108 ultra-short stories: 96 character-related stories and 12 anchor-stories (four stories per character strength/anchor). Every story can be presented independently or be paired with three story-specific “sequels” to produce four different trial types: (1) stories without consequences, (2) stories with positive consequences, (3) stories with mixed consequences, and (4) stories with negative consequences, for a total of 4 × 108 = 432 possible trials (within-subjects design). In every trial, we inquired participants to rate the degree to which they believe that the stories’ agents acted morally correct using a nine-point answer format (anchored at −5 = [they acted] very much morally negatively, 0 = [they acted] neutrally, and +5 = [they acted] very much morally positively). Anchors were the three socially aversive traits of the Dark Triad: Machiavellianism, narcissism, and psychopathy (for an overview, see Paulhus and Williams, 2002; Furnham et al., 2013). The CS-MET (in German) can be found in the CS-MET repository on OSF: https://doi.org/10.17605/OSF.IO/ZGTXQ.

##### Stimuli

Every ultra-short story depicts a different agent engaging in behavior that corresponds to the underlying character strength or Dark Triad trait. Three translated examples are: “A young woman courageously confronts her fear of heights and valiantly scales a climbing wall for the first time in her life” (bravery), “A high school student compassionately tends to his grandfather and assists him with his daily routine” (kindness), and “A young employee has big career goals and is ruthless in achieving them” (Machiavellianism). Notably, expressions such as “high school student” and “young employee” are gender-neutral in English but require specification in German, and hence we assigned half of the agents per strength/Dark Triad trait as female and the other half as male. We likewise diversified the agents’ age per strength/Dark Triad trait by depicting them as either primary or secondary schoolers (6–18 years), young adults (19–34 years), adults (35–64 years), or older adults (more than 65 years). We crafted the stories vis-à-vis the literature and instruments for measuring character strengths and the Dark Triad using Peterson and Seligman’s (2004) handbook, the German VIA-IS’ items (see Ruch et al., 2010), and the German Short Dark Triad’s and Dirty Dozen’s items (see Küfner et al., 2015; Malesza et al., 2019).

The story-specific sequels pertain to either positive, mixed, or negative consequences following the agents’ actions. Translated examples for the previously depicted story on kindness are “The student is cherished by his grandfather and is bequeathed a large portion of his patrimony” (positive), “The student feels appreciated, but tending to his grandfather sometimes keeps him very busy, and he has trouble with his coursework” (mixed), and “The student has an accident while carrying the groceries and has to use crutches for the next 4 weeks” (negative). Half of the sequel stories per strength pertained to consequences for the actors themselves (such as in the examples), while the other half pertained to consequences for others.

##### Pretests and data preparation

Four members of the positive psychology research group at the (University of Zurich) pretested the CS-MET: they provided feedback on the stories’ clarity, whether they did indeed pertain to the character strength/Dark Triad trait in question, and whether they may unintentionally touch on more than one strength/Dark Triad trait, and we amended some of the stories accordingly^3^. In every story that pertained to character strengths, the experts rated the agent’s actions as at least marginally morally positive (*Min* = +1). Overall, the expert ratings ranged from 1.23 (creativity) to 3.88 (fairness) with *Mdn* = 1.88. Next to fairness, the other top five strengths were kindness (3.38), honesty (3.13), and judgment and gratitude (both 3.00).

Additionally, two student assistants completed the full CS-MET and provided feedback on their experience and the elapsed time. They stated that the instructions were clear and that they could readily rate all stories, but that the CS-MET alone took them more than 1 h to complete, which strained their vigilance and motivation. Accordingly, we decided to decrease the individual burden by inquiring participants to only work on a randomly selected portion of the CS-MET’s trials. This approach corresponds to Revelle et al. (2017) SAPA procedure, who showed that data that includes procedurally missing values (missing completely at random; MCAR) can still produce reliable mean-level statistics without the need to present the whole item set. We hence sampled eight strengths and one Dark Triad trait completely at random for a total of 9 (strengths/Dark Triad trait) × 4 (stories) × 4 (trial types) = 144 trials for every participant. In other words, every participant rated stories that correspond to *eight* strengths and *one* Dark Triad trait across all four trial types. In order to further avoid the loss of vigilance and motivation, we administered the CS-MET in two blocks: In the first block, participants appraised their preselected 36 stories without consequences, and in the second block, they appraised the 108 stories with consequences. The trials were randomized within both blocks, and participants could take two breaks: one after the first block and another one after appraising half of the trials of the second block.

For every trial type, participants’ ratings were aggregated across the corresponding four stories, resulting in 9 (strengths/Dark Triad trait) × 4 (trial types) = 36 ratings per participant. The ratings were subsequently aggregated across participants to obtain sample means, standard deviations, and 95% confidence intervals (with Bonferroni correction) of the means for every strength/Dark Triad trait. In the final sample, the stories’ Cronbach’s alpha per character strength ranged from 0.54 (Prudence) to 0.84 (Curiosity) with a median internal consistency of 0.75, and the Dark Triad traits’ Cronbach’s alphas were 0.64 (Machiavellianism), 0.61 (narcissism), and 0.65 (psychopathy).

#### Character Strengths

We used the VIA Inventory of Strengths [VIA-IS: Peterson and Seligman, 2004; German adaptation by Ruch et al. (2010)] to assess the VIA classification’s 24 character strengths (e.g., judgment: “I always examine both sides of an issue”). The VIA-IS formally comprises 240 items (10 items per strength) and uses a five-point answer format (1 = very much unlike me to 5 = very much like me). We also used Revelle et al.s’ (2017) SAPA procedure and sampled 120 items completely at random for every participant. We presented these items in four blocks that each comprised 30 items. In this study, Cronbach’s alpha ranged from 0.60 (Teamwork) to 0.90 (Spirituality) with a median internal consistency of 0.78.

### Data Analysis

We conducted the analyses within the R statistical computing environment (R Core Team, 2019) using Revelle’s (2019) psych package. As we did not formulate hypotheses about the interaction of particular character strengths’ moral evaluations with different consequences following strength-related behavior, we only inspected the corresponding main effects. However, the results of a 27 (character strengths + Dark Triad traits) × 4 (consequences) repeated measures ANOVA/mixed-effects analysis can still be found in the Supplementary Knitr report (see Xie, 2020) to this publication, as can be the input and output of our analyses in general.

In correspondence to Peterson and Seligman’s (2004) criterion, we considered the aggregated ratings for stories without consequences to depict the general moral evaluation of character strengths (“even in the absence of obvious beneficial outcomes”, p. 19). We began by describing our findings for this general moral evaluation and contrasted it from that for stories with positive consequences. Next, we inspected the aggregated ratings for stories with mixed and negative consequences and used Conway and Gawronski’s (2013) process dissociation approach to compute inclinations toward deontology and consequentialism. Conway and Gawronski’s (2013) approach is based on Greene’s (e.g., 2007) dual-process theory and allows for computing separate scores for both inclinations by contrasting evaluations in “congruent” scenarios (in which both inclinations produce *similar* ratings; this should correspond to stories with positive consequences) from those in “incongruent” scenarios (in which both inclinations produce *opposing* ratings; this should correspond to stories with negative consequences). Finally, we examined the relationships of the general moral evaluation with the resulting scores and with character strengths using regression and correlation analysis.

## Results

### Participants Generally Recognize Every Strength as Positively Morally Valued, Even in the Absence of Positive Outcomes

**FIGURE 1:**
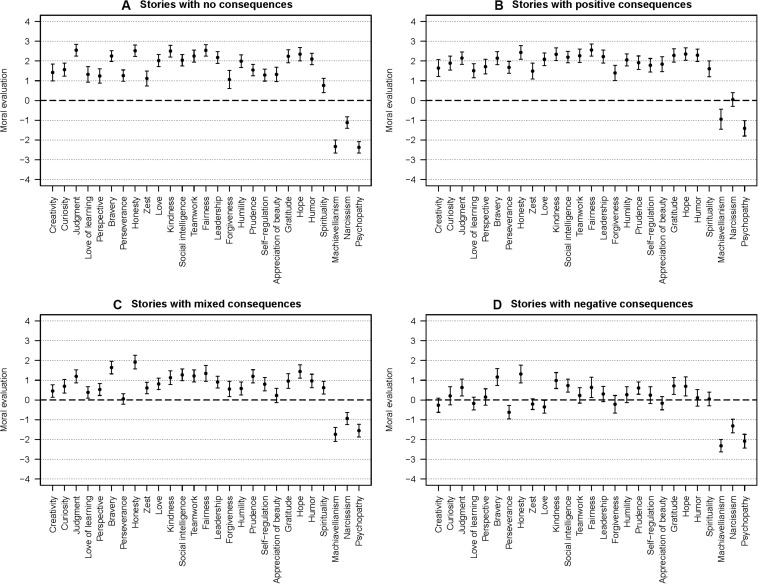
Means and 95% confidence intervals (with Bonferroni correction) of the Character Strengths’ Moral Evaluation Task’s (CS-MET) ratings as functions of the 24 VIA character strengths and the three Dark Triad traits. The panels depict the ratings for the different trial types: **(A)** stories without consequences, **(B)** stories with positive consequences, **(C)** stories with mixed consequences, and **(D)** stories with negative consequences. *N* = 230.

The CS-MET’s overall results are depicted in Figure 1. Figure 1A shows that participants generally recognized every character strength as positively morally valued when the stories were presented without consequences. Conversely, the three Dark Triad traits were generally perceived as negatively morally valued, which can be considered a successful manipulation check. All ratings were significantly different from zero (neutral), but there were notable differences in the effect sizes^4^. We assigned them to one of four tiers according to which thresholds of the scale were or were not entailed by their confidence intervals. Five strengths were rated higher than +2 and were labeled *very positive*: Judgment, honesty, kindness, fairness, and hope. Remarkably, these strengths (except for hope) correspond to the top five strengths that have been identified by the small sample of experts in the pretest. Eight strengths entailed +2 and were labeled *markedly positive*: Bravery, love, social intelligence, teamwork, leadership, humility, gratitude, and humor. Two strengths were rated higher than +1 and were labeled *positive*: Curiosity and prudence. Nine strengths entailed +1 and were labeled *slightly positive*: Creativity, love of learning, perspective, perseverance, zest, forgiveness, self-regulation, appreciation of beauty, and spirituality. Notably, four strengths that have previously been discussed as value-added were rated slightly positive (love of learning, perseverance, zest, and appreciation of beauty), one was rated positive (curiosity), and two were rated markedly positive (humility and humor).

Figure 1B shows that positive consequences following the agents’ actions resulted in a similar pattern and only marginal changes to the average evaluation: the profile correlation was *r*_AB_ = 0.99, and hence the strengths’ rank order can be considered practically equivalent. The average evaluations were also comparable with *M*_A_ = 1.81, 95% CI [1.52, 2.09] and *M*_B_ = 1.99, 95% CI [1.82, 2.16], which indicates that positive consequences generally did not yield more positive evaluations. We concluded that every strength satisfied Peterson and Seligman’s (2004) criterion: Every strength was positively morally valued, and positive consequences did neither account for nor increase this effect by a notable degree. However, there were differences in moral evaluations across strengths, which indicates that some strengths are valued more strongly than others.

### Different Consequences Following Strength-Related Behavior Influence Moral Evaluations and Indicate Differences in Inclinations Toward Deontology and Consequentialism

The other panels show that mixed (Figure 1C) and negative (Figure 1D) consequences following the agents’ actions also resulted in similar patterns, but in more changes to the average evaluations: the profile correlations were *r*_AC_ = 0.95, and *r*_AD_ = 0.92, but participants generally rated the agents’ actions less positively when they were followed by mixed consequences (*M*_C_ = 0.90, 95% CI [0.66, 1.13]) and the least positively when they were followed by negative consequences (*M*_D_ = 0.29, 95% CI [0.04, 0.55]): In trials with mixed consequences, participants rated two previously slightly positive strengths neutrally (perseverance and appreciation of beauty). In trials with negative consequences, participants rated 13 previously slightly positive to markedly positive strengths neutrally (creativity, curiosity, love of learning, perspective, zest, teamwork, leadership, forgiveness, humility, self-regulation, appreciation of beauty, humor, and spirituality) and two strengths negatively (perseverance and love). These include all strengths that have previously been discussed as value-added.

**FIGURE 2:**
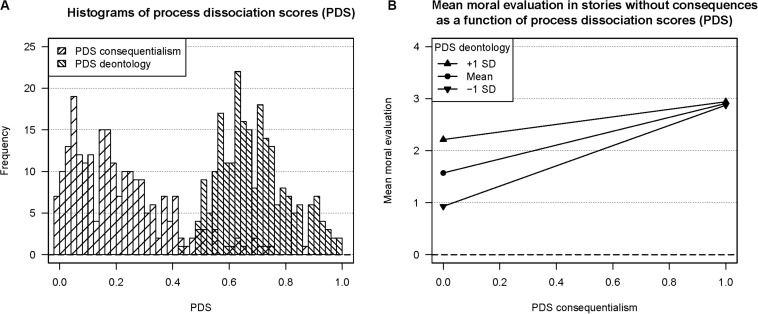
**(A)** Histograms of process dissociation scores (PDS) for consequentialism and deontology. **(B)** Results of the linear regression analysis of the PDS on the mean moral evaluation in the Character Strength’s Moral Evaluation Task’s (CS-MET) stories without consequences: *Mean moral evaluation = –2.15* + *4.87 × PDS consequentialism + 5.39 × PDS deontology – 5.11 × PDS consequentialism* × *PDS deontology*. *R^2^_*Adjusted*_ = 0.*60; *N* = 230.

Based on Conway and Gawronski’s (2013) process dissociation approach, we computed participants’ deontological and consequentialist inclinations by contrasting their ratings in stories with positive consequences and such with negative consequences. In the language of process dissociation, stories with positive consequences should map onto congruent trials because both deontological and consequentialist inclinations should result in positive moral evaluations (assuming that there is indeed some deontological value attached to character strengths). Conversely, stories with negative consequences should map onto incongruent trials because deontological inclinations should result in positive moral evaluations, whereas consequentialist inclinations should result in negative moral evaluations. Notably, our data structure differed from that of Conway and Gawronski (2013) in that participants did not just *choose* but additionally *rated the degree* to which the action depicted in the stories was acceptable or unacceptable. Accordingly, we did not compute the percentages of acquiescence but substituted the “raw” moral evaluations into the formulas^5^ to obtaining the process dissociation scores (PDS) of deontology and consequentialism. However, we normed the ratings to the range of [0, 1] to make our PDS more comparable to those of Conway and Gawronski (2013). A histogram of the PDS is depicted in Figure 2A. It shows that participants differed in their inclinations toward deontology and consequentialism, which corresponds to our observation that the average evaluations in trials with mixed and negative consequences were smaller than those in trials with positive or no consequences. We concluded that different consequences following strength-related behavior can influence moral evaluations and that differences in the ratings correspond to differences in the inclinations toward deontology and consequentialism.

### Inclinations Toward Deontology and Consequentialism and Individual Differences in Character Strengths Influence Moral Evaluations

Figure 2B shows the results of a regression model that included PDS deontology, PDS consequentialism, and their interaction as predictors and the mean moral evaluation in stories without consequences as criterion. Surprisingly, both PDS deontology [*b* = 5.39, *t*(226) = 12.43, *p* < 0.001] and PDS consequentialism [*b* = 4.87, *t*(226) = 4.56, *p* < 0.001] emerged as significant predictors, and their significant negative interaction [*b* = −5.11, *t*(226) = −3.56, *p* < 0.001] indicates that they compensate each other at high levels. Taken together, all predictors can explain the share of *R^2^_Adjusted_* = 0.60. We concluded that–unlike in trials with mixed and negative consequences–inclinations toward deontology *and* consequentialism can account for more positive evaluations in the absence of tangible outcomes. This result suggests that character strengths’ moral value is indeed, for the most part, unanimously recognized as long as the consequences of strength-related actions are unmentioned.

Finally, Table 2 shows that individual differences in eight strengths–as measured by the VIA-IS–correlated with more positive ratings in the CS-MET’s respective strength-related stories: for example, individuals who endorsed zest in the VIA-IS also rated it more positively in the CS-MET’s stories with mixed and negative consequences (small to medium positive effects). Further differences pertained to small to medium positive effects for social intelligence (no/positive consequences), fairness (mixed/negative consequences), leadership (no/positive/mixed consequences), humility (no/positive consequences), gratitude (no/positive/mixed consequences), and humor (no consequences), and to medium to large positive effects for spirituality (no/positive consequences). These include three strengths that have previously been discussed as value-added (zest, humility, and humor). Notably, there were no differences pertaining to very positive strengths. We concluded that individual differences in trait character strengths can also influence moral evaluations, but that this effect may not apply to all strengths.

**TABLE 2:** Correlations of character strengths (as measured by the VIA-IS) with their respective moral evaluations in the CS-MET, split across the four trial types.

**Character strength**	**No consequences**	**Positive consequences**	**Mixed consequences**	**Negative consequences**
Creativity	0.15	0.14	0.08	0.03
Curiosity	0.18	0.17	0.15	0.02
Judgment	0.01	0.06	−0.07	−0.03
Love of learning	0.03	−0.21	0.11	0.22
Perspective	0.18	−0.02	−0.03	−0.02
Bravery	0.03	0.11	0.03	−0.07
Perseverance	0.11	−0.01	−0.11	−0.15
Honesty	0.05	−0.09	−0.06	−0.19
Zest	0.24	0.18	0.26*	0.28*
Love	0.22	0.16	0.17	0.09
Kindness	0.16	0.11	0.07	0.03
Social intelligence	0.24*	0.26*	0.10	0.09
Teamwork	0.16	0.19	0.06	0.00
Fairness	0.20	0.14	0.32**	0.24*
Leadership	0.28**	0.30**	0.30**	0.11
Forgiveness	0.15	0.06	0.21	0.20
Humility	0.26*	0.25*	0.17	0.00
Prudence	0.14	−0.08	−0.10	−0.10
Self-regulation	0.04	−0.08	0.07	0.10
Appreciation of beauty	0.07	0.21	−0.02	−0.14
Gratitude	0.28*	0.26*	0.28*	0.20
Hope	0.16	0.07	−0.01	−0.09
Humor	0.28**	−0.05	0.06	0.09
Spirituality	0.44***	0.34**	0.18	0.09

N = 230; *p < 0.05, **p < 0.01, ***p < 0.001.

## Discussion

This account set out to highlight the importance of scrutinizing the criteria for character strengths, and our study sought to take the first step toward empirically testing one of the most defining yet understudied criteria–*morally valued*–in an online experiment. Based on the responses of a German-speaking convenience sample, we can indeed offer first evidence that “(a)lthough strengths can and do produce desirable outcomes, each strength is morally valued in its own right, even in the absence of obvious beneficial outcomes” (Peterson and Seligman, 2004, p. 19). This is arguably the most important result of our study, and it can therefore not be understated that the criterion seems to *stand*–notwithstanding prior ideas about value-added strengths and individual differences in ethical decision making. However, although our results emphasize the criterion’s validity, they also demonstrate that our previous understanding was oversimplified: character strengths moral evaluation seems to come in degrees, and it can be affected by at least three (and presumably several more) critical factors: The character strengths themselves, their consequences, and also individual differences in inclinations toward deontology and consequentialism and in character strengths.

Most notably, participants valued some strengths more strongly than others. In particular, judgment, honesty, kindness, fairness, and hope constituted the highest-valued strengths, and although it was diminished, their positive value could mostly stand even in the face of negative consequences. On the other hand, creativity, love of learning, perspective, perseverance, and zest (among others) were among the lowest-rated strengths, and it is striking that many of them were previously discussed as value-added. Second, mixed (positive *and* negative) and negative consequences generally accounted for less positive and, in some cases, also for negative evaluations, such as in the case of perseverance and love. Using Conway and Gawronski’s (2013) process dissociation approach, we were able to connect differences in the ratings to differences in inclinations toward deontology and consequentialism, which were prevalent and widely spread in the sample. To our surprise, both inclinations, *including* consequentialism (and their interaction), proved to be strong predictors of character strengths’ moral evaluation in the absence of tangible outcomes. This finding can explain why the criterion can stand in an ethically diversified sample, but it also raises the new question of how individuals who put a strong emphasis on positive consequences can arrive at positive evaluations when there are no such consequences. Finally, individual differences in character strengths seem also to affect moral evaluations, but this effect only pertained to specific strengths and only in the face of specific consequences: For example, individuals who endorsed spirituality arrived at much more positive evaluations in trials with positive and no consequences, and the same was true for individuals who endorsed fairness in trials with mixed and negative outcomes.

Our account demonstrates that reinstalling research into the criteria is useful because it contributes to substantiating character strengths’ theoretical foundation, thus helping us understand what character is. However, it also highlights that working with the criteria produces many more questions that require further attention. Regarding *morally valued*, these questions primarily pertain to why some strengths are valued more positively than others, how consequentialism can lead to more positive evaluations in the absence of tangible outcomes, and–maybe most importantly–where we now want to go and how to proceed from here. As our solution to the first problem hinges on the second one, we will begin by explaining why we believe that consequentialism can yield more positive evaluations in the absence of positive outcomes and address the remaining questions after that. However, it is important to note that more theoretical discussion and empirical research will be needed to answer these questions definitively, and we can hence only provide careful and preliminary explanations to our findings.

### Consequentialism Accounts for More Positive Evaluations Because of an Assumed Connection of Character Strengths With Positive Consequences

As we have stated in the introduction to our account, there is an abundance of studies into character strengths’ positive outcomes, such as well-being (e.g., Park et al., 2004a; Wagner et al., 2019), work performance and academic achievement (e.g., Park and Peterson, 2006; Littman-Ovadia and Lavy, 2016), and resilience (e.g., Peterson et al., 2006; Martínez-Martí and Ruch, 2017). We can now look back on more than 15 years of research into such positive outcomes, and additionally on even more years of research into specific strengths, such as kindness/prosocial behavior (e.g., Batson, 2012) and hope/optimism (e.g., Snyder et al., 2005). Beyond systematic investigations and their presumable influence on laypersons’ understanding of character, we can assume that personal experiences, contemporary media, and folkloric knowledge (among other factors) will have engendered the stereotype that *character accounts for positive consequences*–at least until you are convinced otherwise. Indeed, studies such as Biswas-Diener’s (2006) into the recognition and desirability of character strengths among the Kenyan Maasai or Inughuit in Northern Greenland, or more recent studies by Ruch et al. (2019) and Giuliani et al. (2020) into their perceived fulfillment and virtuousness demonstrate that individuals strongly connect character strengths with such positive outcomes. Accordingly, we can assume that participants who were driven by consequentialism valued character strengths more positively because they assumed them to account for some positive consequences. In other words, they may have believed that positive consequences were *implied* even when they were not mentioned in the story. Indeed, this explanation can fit well into Greene’s (e.g., 2007) dual-process theory of moral judgment because participants seem to have relied more on spontaneous intuitions than on rational processing when giving their rating–else the principle of consequentialism would have dictated to reject character strengths’ moral value. However, it also raises the question whether, under other circumstances, for example when given abundant time and resources (see Conway and Gawronski, 2013; Gawronski and Beer, 2017), individuals would arrive at different judgments, thus again challenging the validity of Peterson and Seligman’s (2004) criterion.

Irrespective of this question, the considerations above suggest that the criterion’s current wording is slightly misleading: although deontologists may subscribe to the notion that character strengths are morally valued “(…) in the absence of obvious beneficial outcomes” (Peterson and Seligman, 2004, p. 19), consequentialists would certainly not. They would either assume that there is some implicit connection with positive outcomes–which seems to be the case in our sample–or they would not and thus reject the criterion. In any case, the connection with such outcomes is critical, and although it may not be immediately obvious to the judges themselves, its pertinent role in this explanation makes it obvious to the observer. It would undoubtedly be too early to revise the criterion based on only one study, but we hope that our account can animate more discussion into this issue and spawn new empirical studies that test our or similar hypotheses. To this end, we believe that either of two approaches may prove to be especially fruitful: Experimentally priming the connection of character strengths and positive outcomes before administering the CS-MET or using cognitive interviewing (see, e.g., Beatty and Willis, 2007) during administration. For example, a simple experimental design could involve attempting to manipulate this connection by providing some participants with a popular review on character strengths’ positive outcomes. In contrast, the remainder would be provided with a review on the “dark side” of character strengths, such as strength underuse, overuse, and their correlations with psychopathology (see Freidlin et al., 2017; Littman-Ovadia and Freidlin, 2020). If the manipulation was successful, this could result in the first group rating character strengths generally more positively than the second group, which would support our hypothesis. On the other hand, cognitive interviewing could involve inquiring participants (especially those with inclinations toward consequentialism) to explain how they arrived at their specific judgments at the moment they are giving it. This would require participants to be able to have some cognitive access to their processing–which may or may not be the case–but it could result in more unbiased findings that either substantiate, extend, or challenge our hypothesis.

### Why Are Some Strengths Valued More Positively Than Others?

#### Some Strengths Are Valued More Positively Because of an Assumed Stronger Connection With Positive Outcomes

Following our considerations on how consequentialism and deontology may have driven participants’ moral evaluations, we can offer two tentative explanations on why some strengths were consistently valued more positively than others, and how the concept of value-added strengths may fit into this picture. First, if our hypothesis was correct and consequentialists generally valued character strengths due to their assumed connection with positive outcomes, strengths that sustain stronger connections and those that produce more or more important positive outcomes might also yield more positive evaluations. Indeed, this hypothesis is supported by findings on strengths such as hope, which typically yields stronger relationships with well-being than most other strengths (e.g., Park et al., 2004a; Wagner et al., 2019) or bravery, which emerged as one of the most potent correlates of resilience (e.g., Peterson et al., 2006; Martínez-Martí and Ruch, 2017). However, there are also several findings that seem to contradict this hypothesis, such as those on perseverance, which was among the lowest-rated strengths but also among the strongest correlates of performance and academic achievement (e.g., Park and Peterson, 2006; Littman-Ovadia and Lavy, 2016). Overall, there is only selective overlap between our results and those reported in the literature, and as long as we do not have reason to suspect that we are looking at the wrong outcomes, our hypothesis can only apply if character strengths’ moral value hinges more strongly on the *assumed* qualities of the connection than on the qualities that we can find in correlational studies.

This would make the experimental design that we have outlined above even more interesting: Instead of priming character strengths’ connection with positive outcomes in general, we could attempt to prime only some selected strengths’ connection and explore whether this also leads to more positive evaluations in the targeted strengths (but not in those that were untargeted). Alternatively, we may investigate whether an individual’s knowledge of research findings into character strengths’ specific connections with positive outcomes can strengthen the match between these findings and their ratings. Taken together, it is plausible that the assumed connection between strengths and positive outcomes not only affects *whether* but also *the degree to which* consequentialists perceive character strengths as positively morally valued. In this framework, value-added strengths would correspond to those that are believed to sustain the weakest or the smallest number of connections with important positive outcomes. In this sense, it may be better to speak of *value-at-risk strengths*, as their model value may fail to be recognized if there are occasional negative outcomes. However, due to the incongruence between the literature and our findings, and assuming that educated individuals (such as in our sample) may have a fair understanding of character strengths’ connection with positive outcomes (e.g., that perseverance can contribute to performance) we are inclined to believe that this hypothesis has only little bearing on the ratings. Instead, we believe that the following, deontologically grounded hypothesis, primarily drives individuals’ judgments.

#### Some Strengths Are Valued More Positively Because of an Assumed Better Qualification for Universal Principles of Conduct

Earlier, we raised the idea that Peterson and Seligman (2004) may have put particular emphasis on character strengths’ moral integrity in the absence of positive outcomes because they wanted to make an argument for the principle of deontology. This principle can essentially be defined by Kant’s (1785/2007) categorical imperative: “Act only according to that maxim whereby you can, at the same time, will that it should become a universal law.” Consequently, if individuals’ moral evaluations are driven by deontology, and they arrive at very positive evaluations, their evaluations should map onto character strengths’ qualifications for such universal principles of conduct. In other words, individuals who arrive at particularly positive ratings for specific character strengths may believe that the actions associated with these strengths can universalize across a number–if not the majority–of different scenarios in which they can find themselves in. For example, as participants have assigned very positive evaluations to judgment and honesty, they may believe that it would be best if they would always act with practical wisdom, considering all facets and perspectives when attempting to make a decision and to always be honest toward themselves and others. In contrast, as creativity and zest were rated considerably less positive, participants may believe that they can usually let their creativity flow and approach life enthusiastically, but that there is also a number of situations in which such actions would be ill-advised. Remarkably, this would make the VIA classification not only a catalog of human strengths but–presumably by coincidence–also one of such deontological principles.

This is undoubtedly a bold claim, and due to its potential theoretical repercussions, it will require sensitive discussion and strong empirical substantiation. Most notably, and in line with Greene’s dual-process model (e.g., Greene, 2007; Conway and Gawronski, 2013), we can assume that many individuals will not *reason* in the strictest sense but instead resort to stereotypes when rating character strengths’ moral value: They may not ponder on character strengths’ qualification to universalize inasmuch as they decide according to their affective responses and intuitions. Still, it is possible that character strengths’ “universalizability” may sensibly shape such stereotypes, be it by positive individual experiences, cultural norms, or even by what Peterson and Seligman (2004, p. 13) call “an evolutionary process that selected for these aspects of excellence as means of solving the important tasks necessary for survival of the species”. Taken together, it may be the degree to which character strengths qualify for general principles of conduct that explains which strengths are particularly valued by deontologists. In this framework, value-added strengths would correspond to those that are believed to qualify as guiding principles only for a lesser number of scenarios in which individuals can find themselves in. We may speculate that it was this lack of universalizability (“positively valued, but only under specific circumstances”) that led other researchers to believe that such value-added strengths can exist. Future studies may choose to inquire participants whether they believe that specific strengths can universalize to such principles and correlate their responses with their ratings in the CS-MET. However, as it is unclear to what extent individuals can reason about their decision, this may or may not yield conclusive findings. Above all else, we believe that this hypothesis demands theoretical attention, especially the joint efforts from psychology and philosophy, to develop a model that can conceptually unify our findings.

### Limitations

This study’s results and inferences are subject to a number of limitations that primarily pertain to the characteristics of our sample and our experimental design. First, we chose to recruit a convenience sample to test whether Peterson and Seligman’s (2004) criterion can stand in the face of diverse ethical inclinations and differences in affective, cognitive, and motivational states. The variance in the process dissociation scores of deontology and consequentialism implies that we were able to recruit such an ethically diverse sample, but we have not collected information about participants’ affective, cognitive, and motivational states. Specifically, we have not collected information about their ability and motivation to engage in ethical reasoning prior to giving their ratings instead of resorting to heuristics and stereotypes. It is hence unclear to what extent inclinations toward deontology and consequentialism were the product of such reasoning or of rather intuitive, automatic processing (see Greene, 2007; Conway and Gawronski, 2013). A related issue is that our only indicator of the CS-MET’s reliability are the stories’ internal consistencies and that we have not collected data on the ratings’ stability. It is hence unclear whether participants would arrive at the same ratings when questioned again and whether supposed fluctuations should be considered a characteristic of a lack of reliability or of differences in participants’ processing. In any case, the CS-MET’s reliability and what variation in the ratings means should be subject to further discussion and scrutiny. Moreover, our sample mainly comprised relatively young, female individuals who were highly educated and were presumably raised in a WEIRD society (see Henrich et al., 2010). We hope that our account can spawn more interest in scrutinizing the criteria–particularly *morally valued*–and that our experimental approach will be adopted in a study that recruits their sample from a different cultural background. This would also contribute to testing our findings’ cultural invariance: specifically, if character strengths proved to map onto general deontological principles, we would hope that future research would also explore whether such principles can universalize across cultures in a fashion that corresponds to character strengths’ criterion of ubiquity (see Park, 2018).

Second, it cannot be ruled out that our findings were partially produced by our experimental design and thus rather reflect methodological issues instead of differences in moral evaluations. The within-subjects design allowed participants to indirectly rate the same strengths across different trials, but it may have also introduced artificial variance due to this sequencing. For example, stories with negative consequences may have been perceived as much more negative in comparison to stories with positive or no consequences, thus accounting for bias in moral evaluations. We used the three socially aversive traits of the Dark Triad in order to avoid that participants feel required to produce variance in their ratings. However, this may have also accounted for stronger distinctions between evaluations of such traits and those of character strengths. Future research may choose to use instead what Peterson and Seligman (2004, p. 299) called “(…) talent(s) or abilit(ies) that fall outside the moral realm” (e.g., general intelligence, athletic ability, or perfect pitch) as anchors. However, we suggest not using personality traits such as the Big Five: Such traits will presumably also be positively morally valued because they conceptually include many character-related traits. This can be seen in Allport and Odbert’s (1936, pp. 38–171) adjective lists, which also include terms such as “honest,” “humorous,” and “modest,” and in McGrath et al.’s (2020) study into the overlap between the VIA classification, the Big Five, and the HEXACO model.

Third, it is unclear whether a certain degree of social desirability may have influenced participants’ ratings. Specifically, it may be assumed that participants rated certain character strengths more positively because they thought that this was expected from them and not because they themselves believed that the strengths hold inherent moral value. As the experiment was administered online and participants submitted their ratings anonymously, we do not believe that they felt particularly required to respond according to such norms. However, previous research using the American English (Peterson and Seligman, 2004) and the German (Ruch et al., 2010) VIA-IS showed that some strengths were significantly (albeit weakly) associated with measures of social desirability (e.g., prudence, honesty, humility in a German-speaking sample). Accordingly, future research may choose to test whether such effects can also be found in the CS-MET and–if they could be found–what would make participants feel required to modify their ratings.

Finally, as stories were initially presented without consequences, and participants were not explicitly alluded to the experimental design, they may not have considered that the agents’ actions could also produce negative consequences. Indeed, our stories may be criticized for not only implicitly but also explicitly stating that the agents’ actions accounted for some positive consequences. In our exemplary story for bravery–“A young woman courageously confronts her fear of heights and valiantly scales a climbing wall for the first time in her life”–successfully scaling a climbing wall could be perceived as a positive consequence in itself. This is a conceptual problem because character strengths and such inherent consequences cannot be split without stripping the strength of its meaning. For example, Peterson and Seligman’s (2004, p. 29) consensual definition of bravery entails “(…) speaking up for what is right even if there is opposition (…)”, and speaking up may or may not already be perceived as such a positive consequence (e.g., of overcoming fear). It can be assumed that there are individual differences in the degree to which participants expected further consequences or noticed that this might not be the end of the story, and such differences may have also biased our results. Future research may choose to allude participants to the experimental design and explain that they will first rate scenarios without consequences, followed by scenarios with consequences, but this might arguably also account for greater differences between these two blocks.

## Conclusion

This account shows that scrutinizing the criteria for character strengths is useful because it helps us understand what character is and what sets it apart from other individual differences. Presumably, most readers were familiar with the criteria and had “nodded them off,” but we suspect that few would have subscribed to the notion that character strengths may qualify for deontological principles of conduct or that their moral value may also be grounded in their implicit connection with positive outcomes. Our study focused on character strengths’ assumed moral evaluation, which we could provide first evidence on. However, the most important message is arguably that research into the criteria is generally *possible*, and we hope that our impetus will animate similar endeavors in exploring the implications of observing individuals who endorse certain strengths to a striking degree (*paragons*) or who can inspire other individuals instead of belittling them (*does not diminish others*).

Investigating the validity of the criteria is not without peril because it means that character strengths can fail them, thus casting a certain degree of doubt on the classification as a whole. However, without this discussion and empirical studies, we cannot know, and the classification cannot proceed in a fashion Peterson and Seligman (2004, p. 31) envisioned when they wrote: “(…) we expect [the classification] to change in the years to come, as theory and research concerning character strengths proceed. (…) We anticipate that our classification of strengths will (…) evolve, by adding or deleting specific strengths of character, by combining those that prove redundant, by reformulating their organization under core virtues, and by more systematically evaluating them vis-à-vis our (…) criteria.”

## Data Availability Statement

The datasets generated in this study can be found in online repositories. The names of the repository/repositories and accession number(s) can be found below: https://doi.org/10.17605/OSF.IO/ZGTXQ.

## Ethics Statement

Ethical review and approval was not required for the study on human participants in accordance with the local legislation and institutional requirements. The patients/participants provided their written informed consent to participate in this study.

## Author Contributions

AS and WR conceptualized and designed the study. AS collected the data, performed the statistical analysis, and wrote the first draft of the manuscript. Both authors contributed to manuscript revision and read and approved the submitted version.

## Conflict of Interest

WR is a Senior Scientist for the VIA Institute on Character, which holds the copyright to the VIA Inventory of Strengths.

The remaining author declares that the research was conducted in the absence of any commercial or financial relationships that could be construed as a potential conflict of interest.

## Footnotes

1 Gordon Allport served as president of the American Psychological Association in 1939 and is considered one of the founding figures of personality psychology.

2 Ubiquity is recognized as another criterion for character strengths (see Park, 2018). For an overview of the research on this criterion, see Ruch and Stahlmann (2019).

3 An example for such an amended story for perseverance is: “A student assistant shows his persistence and tenacity by continuing to work while others have already resigned.” The original story was flagged for additionally touching on kindness: “A student assistant shows his persistence and tenacity by trying to manage his coworkers’ tasks while they have already resigned”.

4 These findings generally apply to both character strengths and Dark Triad traits, but in line with this study’s aims, the statistics described in the following paragraphs only involve character strengths.

5 Following Conway and Gawronski (2013, pp. 219–220), our formulas were: *PDS consequentialism = mean moral evaluation* in *trials with positive consequences – mean moral evaluation* in *trials with negative consequences* and *PDS deontology = mean moral evaluation* in *trials with negative consequences/(1 − PDS consequentialism)*. Our calculations can also be found in the Supplementary Knitr document to this publication.
