# Supplementary material for: Scrutinizing the Criteria for Character Strengths: Laypersons Assert That Every Strength Is Positively Morally Valued, Even in the Absence of Tangible Outcomes
Source: Front Psychol. 2020 Sep 30;11:591028. doi: 10.3389/fpsyg.2020.591028 (PMC7554639; doi:10.3389/fpsyg.2020.591028)
Supplement: Supplementary file 1 [file Data_Sheet_1.zip › Supplementary Materials.html]

Character strengths are positively morally valued, even in the absence of tangible outcomes: Evidence from an online experiment: Input and output


# Character strengths are positively morally valued, even in the absence of tangible outcomes: Evidence from an online experiment: Input and output

#### A. G. Stahlmann & W. Ruch

#### July 24th, 2020

```
### Preparation ####
## Load packages
library(psych)
library(corrplot)
```

```
## corrplot 0.84 loaded
```

```
library(reshape2)
library(lsmeans)
```

```
## Loading required package: emmeans
```

```
## Welcome to emmeans.
## NOTE -- Important change from versions <= 1.41:
##     Indicator predictors are now treated as 2-level factors by default.
##     To revert to old behavior, use emm_options(cov.keep = character(0))
```

```
## The 'lsmeans' package is now basically a front end for 'emmeans'.
## Users are encouraged to switch the rest of the way.
## See help('transition') for more information, including how to
## convert old 'lsmeans' objects and scripts to work with 'emmeans'.
```

```
library(lme4)
```

```
## Loading required package: Matrix
```

```
## Load data
load("~/Dropbox/AS/Schreiben/Morally_valued/Final/CS-MET.RData")

## Overview
# MV = Complete dataset
# VIA = VIA-IS scales

## Compute means across the four situations
lab = c("Creativity", "Curiosity", "Judgment", "Love of learning", "Perspective", "Bravery",
        "Perseverance", "Honesty", "Zest", "Love", "Kindness", "Social intelligence",
        "Teamwork", "Fairness", "Leadership", "Forgiveness", "Humility", "Prudence",
        "Self-regulation", "Appreciation of beauty", "Gratitude", "Hope", "Humor", "Spirituality",
        "Machiavellianism", "Narcissism", "Psychopathy")

# No outcome
LCS = NULL
for(i in 1:27) {
  LCS[[i]] = c((4*(i-1)+1):(4*i))
}
S.NO.temp = MV[, c(268:(268+107))]
S.NO = NULL
for(i in 1:27) {
  AB = rowMeans(S.NO.temp[, LCS[[i]]], na.rm = T)
  S.NO = cbind(S.NO, AB)
}
S.NO[is.nan(S.NO)] = NA
colnames(S.NO) = lab
S.NO = S.NO - 5

# Positive outvome
S.PO.temp = MV[, c(376:(376+107))]
S.PO = NULL
for(i in 1:27) {
  AB = rowMeans(S.PO.temp[, LCS[[i]]], na.rm = T)
  S.PO = cbind(S.PO, AB)
}
S.PO[is.nan(S.PO)] = NA
colnames(S.PO) = lab
S.PO = S.PO - 5

# Mixed outcome
S.MI.temp = MV[, c(484:(484+107))]
S.MI = NULL
for(i in 1:27) {
  AB = rowMeans(S.MI.temp[, LCS[[i]]], na.rm = T)
  S.MI = cbind(S.MI, AB)
}
S.MI[is.nan(S.MI)] = NA
colnames(S.MI) = lab
S.MI = S.MI - 5

# Negative outcome
S.NE.temp = MV[, c(592:(592+107))]
S.NE = NULL
for(i in 1:27) {
  AB = rowMeans(S.NE.temp[, LCS[[i]]], na.rm = T)
  S.NE = cbind(S.NE, AB)
}
S.NE[is.nan(S.NE)] = NA
colnames(S.NE) = lab
S.NE = S.NE - 5


### 3.1 Participants generally recognize every strength as positively morally valued AND 3.2    Different consequences following strength-related behavior influence moral evaluations ####
## CS-MET's overall results
splot = function(x, MN = "Title") {
  M = colMeans(x, na.rm = T)
  SD = apply(x, 2, sd, na.rm = T)
  N = nrow(x)-apply(x, 2, function(x) sum(is.na(x)))
  ME = qt(1-.025/27*4, df = nrow(MV)-1)*SD/sqrt(N)
  LCI = M-ME
  UCI = M+ME
  OP = par(bg = "white", mar = c(10, 4, 2, 1)+.1)
  plot(NA, axes = F, xlim = c(1, 27), ylim = c(-4, 4), xlab = "", ylab = "Moral evaluation", main = MN)
  arrows(c(1:27), LCI, c(1:27), UCI, angle = 90, code = 3, length = .025, lwd = 1)
  points(c(1:27), colMeans(x, na.rm = T), pch = 16, cex = .8)
  axis(1, 1:27, lab, las = 2)
  axis(2, seq(-4, 4, 1), las = 2)
  abline(h = c(-4, -3, -2, -1, 1, 2, 3, 4), lty = 2, lwd = .45)
  abline(h = 0, lty = 5, lwd = 1.45)
  box(lwd = 1.25)
}

# pdf(file = "Rplot.pdf", width = 14, height = 14, paper = "a4r")
par(mfrow = c(2, 2), mar = c(4, 4, 4, 2)+.1)
splot(S.NO, "(A): Stories with no consequences")
splot(S.PO, "(B): Stories with positive consequences")
splot(S.MI, "(C): Stories with mixed consequences")
splot(S.NE, "(D): Stories with negative consequences")
```

```
# dev.off()

## Profile correlations
M.NO = colMeans(S.NO, na.rm = T)
M.PO = colMeans(S.PO, na.rm = T)
M.MI = colMeans(S.MI, na.rm = T)
M.NE = colMeans(S.NE, na.rm = T)
corr.test(cbind(M.NO, M.PO, M.MI, M.NE))
```

```
## Call:corr.test(x = cbind(M.NO, M.PO, M.MI, M.NE))
## Correlation matrix 
##      M.NO M.PO M.MI M.NE
## M.NO 1.00 0.99 0.95 0.92
## M.PO 0.99 1.00 0.94 0.91
## M.MI 0.95 0.94 1.00 0.97
## M.NE 0.92 0.91 0.97 1.00
## Sample Size 
## [1] 27
## Probability values (Entries above the diagonal are adjusted for multiple tests.) 
##      M.NO M.PO M.MI M.NE
## M.NO    0    0    0    0
## M.PO    0    0    0    0
## M.MI    0    0    0    0
## M.NE    0    0    0    0
## 
##  To see confidence intervals of the correlations, print with the short=FALSE option
```

```
## Mean differences across consequences
N = 24

# No outcome
M.NO.M = mean(M.NO[c(1:24)])
M.NO.S = sd(M.NO[c(1:24)])
M.NO.ME = qt(1-.025/4, df = nrow(MV)-1)*M.NO.S/sqrt(N)
paste(round(M.NO.M-M.NO.ME, 2), " < ", round(M.NO.M, 2), " < ", round(M.NO.M+M.NO.ME, 2))
```

```
## [1] "1.52  <  1.81  <  2.09"
```

```
# Positive outcome
M.PO.M = mean(M.PO[c(1:24)])
M.PO.S = sd(M.PO[c(1:24)])
M.PO.ME = qt(1-.025/4, df = nrow(MV)-1)*M.PO.S/sqrt(N)
paste(round(M.PO.M-M.PO.ME, 2), " < ", round(M.PO.M, 2), " < ", round(M.PO.M+M.PO.ME, 2))
```

```
## [1] "1.82  <  1.99  <  2.16"
```

```
# Mixed outcome
M.MI.M = mean(M.MI[c(1:24)])
M.MI.S = sd(M.MI[c(1:24)])
M.MI.ME = qt(1-.025/4, df = nrow(MV)-1)*M.MI.S/sqrt(N)
paste(round(M.MI.M-M.MI.ME, 2), " < ", round(M.MI.M, 2), " < ", round(M.MI.M+M.MI.ME, 2))
```

```
## [1] "0.66  <  0.9  <  1.13"
```

```
# Negative outcome
M.NE.M = mean(M.NE[c(1:24)])
M.NE.S = sd(M.NE[c(1:24)])
M.NE.ME = qt(1-.025/4, df = nrow(MV)-1)*M.NE.S/sqrt(N)
paste(round(M.NE.M-M.NE.ME, 2), " < ", round(M.NE.M, 2), " < ", round(M.NE.M+M.NE.ME, 2))
```

```
## [1] "0.04  <  0.29  <  0.55"
```

```
### 3.3 Individual differences in character and philosophical leaning influence moral evaluations ####
## Correlations of VIA character strengths with the CS-MET's ratings
C1 = corr.test(VIA, S.NO[, c(1:24)], adjust = "none")
C2 = corr.test(VIA, S.PO[, c(1:24)], adjust = "none")
C3 = corr.test(VIA, S.MI[, c(1:24)], adjust = "none")
C4 = corr.test(VIA, S.NE[, c(1:24)], adjust = "none")

CT = rbind(diag(C1$r), diag(C2$r), diag(C3$r), diag(C4$r))
PT = rbind(diag(C1$p), diag(C2$p), diag(C3$p), diag(C4$p))
colnames(CT) = colnames(PT) = colnames(S.NO)[1:24]
rownames(CT) = rownames(PT) = c("No consequences", "Positive consequences", "Mixed consequences", "Negative consequences")

dev.off()
```

```
## null device 
##           1
```

```
corrplot(CT, method = "pie", p.mat = PT)
CT
```

```
##                       Creativity  Curiosity    Judgment Love of learning
## No consequences       0.14576565 0.17587392  0.01464045       0.03400204
## Positive consequences 0.14373963 0.17165268  0.05532532      -0.20536879
## Mixed consequences    0.08205985 0.14855814 -0.07139255       0.11264123
## Negative consequences 0.03232792 0.02499779 -0.03200933       0.22442329
##                       Perspective     Bravery Perseverance     Honesty
## No consequences        0.17708874  0.02894676  0.114972313  0.04941673
## Positive consequences -0.02383633  0.11158559 -0.008401848 -0.09281350
## Mixed consequences    -0.03137399  0.03123202 -0.107311127 -0.06210923
## Negative consequences -0.02055139 -0.07472081 -0.146870250 -0.18510836
##                            Zest       Love   Kindness Social intelligence
## No consequences       0.2419988 0.22251487 0.15596801          0.24311211
## Positive consequences 0.1762279 0.15993019 0.11044606          0.26289744
## Mixed consequences    0.2628216 0.17106362 0.07022039          0.09854203
## Negative consequences 0.2801118 0.08634569 0.03295520          0.08586489
##                           Teamwork  Fairness Leadership Forgiveness
## No consequences        0.158947912 0.2024936  0.2844760  0.15387173
## Positive consequences  0.193139872 0.1401023  0.2998257  0.06158387
## Mixed consequences     0.060085250 0.3206361  0.2991907  0.21031739
## Negative consequences -0.001389555 0.2417875  0.1113779  0.20406551
##                           Humility    Prudence Self-regulation
## No consequences        0.263544410  0.13876835      0.04416100
## Positive consequences  0.250514173 -0.08133091     -0.08426379
## Mixed consequences     0.169007398 -0.09525366      0.07116762
## Negative consequences -0.003569586 -0.09964094      0.09760829
##                       Appreciation of beauty Gratitude         Hope       Humor
## No consequences                   0.06698596 0.2810846  0.164973443  0.28426726
## Positive consequences             0.20954220 0.2589780  0.074602157 -0.04894382
## Mixed consequences               -0.02228384 0.2756935 -0.005299635  0.06228341
## Negative consequences            -0.14385226 0.2019145 -0.090356909  0.08623663
##                       Spirituality
## No consequences         0.44388354
## Positive consequences   0.33998022
## Mixed consequences      0.18354456
## Negative consequences   0.09320471
```

```
## Variance in philosophical leaning toward deontology and consequentialism
# Compute process dissociation scores (PDS) in a fashion akin to Conway and Gawronski (2013) https://doi.org/10.1037/a0031021
par(mfrow = c(1, 2))

S.NO.M = rowMeans(S.NO[, c(1:24)], na.rm = T)
S.PO.M = rowMeans(S.PO[, c(1:24)], na.rm = T)
S.MI.M = rowMeans(S.MI[, c(1:24)], na.rm = T)
S.NE.M = rowMeans(S.NE[, c(1:24)], na.rm = T)

S.PO.P = (S.PO.M + 4) / 8
S.NE.P = (S.NE.M + 4) / 8

CON = S.PO.P - S.NE.P
DEO = S.NE.P / (1-CON)

HC = hist(CON, 50, plot = F)
HD = hist(DEO, 40, plot = F)
plot(HC, xlim = c(0, 1), ylim = c(0, 25), density = 7.5, angle = 45, main = "(A) Histograms of process dissociation scores (PDS)",
     xlab = "PDS", las = 1)
abline(h = seq(0, 25, 5), lty = 2, lwd = .45)
abline(h = 0, lty = 5, lwd = 1.45)
plot(HD, xlim = c(0, 1), density = 15, angle = -45, add=T)
legend("topleft", c("PDS consequentialism", "PDS deontology"), angle = c(45, -45), density = c(20, 20))
box(lwd = 1.25)

# Regression of PDS on moral evaluations in the absence of outcomes
M.NO = lm(S.NO.M ~ CON*DEO)
summary(M.NO)
```

```
## 
## Call:
## lm(formula = S.NO.M ~ CON * DEO)
## 
## Residuals:
##      Min       1Q   Median       3Q      Max 
## -1.37528 -0.28699 -0.02978  0.29692  2.26211 
## 
## Coefficients:
##             Estimate Std. Error t value Pr(>|t|)    
## (Intercept)  -2.1509     0.2959  -7.270 5.83e-12 ***
## CON           4.8672     1.0683   4.556 8.53e-06 ***
## DEO           5.3894     0.4301  12.531  < 2e-16 ***
## CON:DEO      -5.1133     1.4376  -3.557 0.000457 ***
## ---
## Signif. codes:  0 '***' 0.001 '**' 0.01 '*' 0.05 '.' 0.1 ' ' 1
## 
## Residual standard error: 0.4921 on 226 degrees of freedom
## Multiple R-squared:  0.606,  Adjusted R-squared:  0.6008 
## F-statistic: 115.9 on 3 and 226 DF,  p-value: < 2.2e-16
```

```
NOC = M.NO$coefficients
NO = function(x) {
  SDU = as.numeric(NOC[1] + NOC[2]*x + NOC[3]*(mean(DEO)+sd(DEO)) + NOC[4]*x*(mean(DEO)+sd(DEO)))
  MN = as.numeric(NOC[1] + NOC[2]*x + NOC[3]*mean(DEO) + NOC[4]*x*mean(DEO))
  SDL = as.numeric(NOC[1] + NOC[2]*x + NOC[3]*(mean(DEO)-sd(DEO)) + NOC[4]*x*(mean(DEO)-sd(DEO)))
  return(c(SDL, MN, SDU))
}
L1 = NO(0)
L2 = NO(1)

plot(NA, ylim = c(-4, 4), xlim = c(0, 1), xlab = "PDS consequentialism", ylab = "Mean moral evaluation",
     main = "(B) Mean moral evaluation in stories without consequences\nas a function of process dissociation scores (PDS)", las = 1)
abline(h = c(1, 2, 3, 4), lty = 2, lwd = .45)
abline(h = 0, lty = 5, lwd = 1.45)
segments(0, L1, 1, L2, lwd = 1.25)
points(c(0, 1), c(L1[2], L2[2]), pch = 21, bg = 1)
points(c(0, 1), c(L1[1], L2[1]), pch = 25, bg = 1)
points(c(0, 1), c(L1[3], L2[3]), pch = 24, bg = 1)
legend("topleft", legend = c("+1 SD   ", "Mean   ", "-1 SD"), lty = 1, lwd = 1.25, pch = c(24, 21, 25), pt.bg = 1,
       title = "PDS deontology")
box(lwd = 1.25)


### ANOVA/mixed-effects analyis ####
## Linear mixed-effects modeling is used because of the NAs that the SAPA procedure produced.
S.NO.L = data.frame(melt(S.NO), rep("no outcome", 230))
S.PO.L = data.frame(melt(S.PO), rep("positive outcome", 230))
S.MI.L = data.frame(melt(S.MI), rep("mixed outcome", 230))
S.NE.L = data.frame(melt(S.NE), rep("negative outcome", 230))
colnames(S.NO.L) = colnames(S.PO.L) = colnames(S.MI.L) = colnames(S.NE.L) = c("ID", "Strength", "Score", "Condition")

SL = rbind(S.NO.L, S.PO.L, S.MI.L, S.NE.L)
SL$ID = factor(SL$ID)
SL$Strength = factor(SL$Strength)
SL$Condition = factor(SL$Condition)

GLM = lmer(Score ~ Strength*Condition + (1|ID), data = SL)
anova(GLM)
```

```
## Analysis of Variance Table
##                    Df Sum Sq Mean Sq   F value
## Strength           26 7707.8  296.45  298.4593
## Condition           3 3419.6 1139.88 1147.5907
## Strength:Condition 78  612.4    7.85    7.9038
```

```
ME1 = emmeans(GLM, list(pairwise ~ Strength), adjust = "tukey")
```

```
## Note: D.f. calculations have been disabled because the number of observations exceeds 3000.
## To enable adjustments, add the argument 'pbkrtest.limit = 8280' (or larger)
## [or, globally, 'set emm_options(pbkrtest.limit = 8280)' or larger];
## but be warned that this may result in large computation time and memory use.
```

```
## Note: D.f. calculations have been disabled because the number of observations exceeds 3000.
## To enable adjustments, add the argument 'lmerTest.limit = 8280' (or larger)
## [or, globally, 'set emm_options(lmerTest.limit = 8280)' or larger];
## but be warned that this may result in large computation time and memory use.
```

```
## NOTE: Results may be misleading due to involvement in interactions
```

```
ME2 = emmeans(GLM, list(pairwise ~ Condition), adjust = "tukey")
```

```
## Note: D.f. calculations have been disabled because the number of observations exceeds 3000.
## To enable adjustments, add the argument 'pbkrtest.limit = 8280' (or larger)
## [or, globally, 'set emm_options(pbkrtest.limit = 8280)' or larger];
## but be warned that this may result in large computation time and memory use.
```

```
## Note: D.f. calculations have been disabled because the number of observations exceeds 3000.
## To enable adjustments, add the argument 'lmerTest.limit = 8280' (or larger)
## [or, globally, 'set emm_options(lmerTest.limit = 8280)' or larger];
## but be warned that this may result in large computation time and memory use.
```

```
## NOTE: Results may be misleading due to involvement in interactions
```

```
IA = emmeans(GLM, list(pairwise ~ Strength*Condition), adjust = "tukey")
```

```
## Note: D.f. calculations have been disabled because the number of observations exceeds 3000.
## To enable adjustments, add the argument 'pbkrtest.limit = 8280' (or larger)
## [or, globally, 'set emm_options(pbkrtest.limit = 8280)' or larger];
## but be warned that this may result in large computation time and memory use.
```

```
## Note: D.f. calculations have been disabled because the number of observations exceeds 3000.
## To enable adjustments, add the argument 'lmerTest.limit = 8280' (or larger)
## [or, globally, 'set emm_options(lmerTest.limit = 8280)' or larger];
## but be warned that this may result in large computation time and memory use.
```

```
ME1$`emmeans of Strength`
```

```
##  Strength               emmean     SE  df asymp.LCL asymp.UCL
##  Creativity              0.764 0.0742 Inf     0.619     0.910
##  Curiosity               1.098 0.0698 Inf     0.961     1.235
##  Judgment                1.610 0.0660 Inf     1.481     1.739
##  Love of learning        0.796 0.0720 Inf     0.655     0.937
##  Perspective             0.882 0.0694 Inf     0.746     1.018
##  Bravery                 1.904 0.0701 Inf     1.767     2.042
##  Perseverance            0.634 0.0666 Inf     0.504     0.765
##  Honesty                 1.994 0.0694 Inf     1.858     2.130
##  Zest                    0.786 0.0733 Inf     0.643     0.930
##  Love                    1.182 0.0712 Inf     1.042     1.322
##  Kindness                1.862 0.0678 Inf     1.729     1.995
##  Social intelligence     1.490 0.0669 Inf     1.359     1.621
##  Teamwork                1.454 0.0675 Inf     1.322     1.586
##  Fairness                1.729 0.0694 Inf     1.593     1.866
##  Leadership              1.310 0.0675 Inf     1.177     1.442
##  Forgiveness             0.691 0.0684 Inf     0.557     0.825
##  Humility                1.339 0.0691 Inf     1.204     1.475
##  Prudence                1.318 0.0709 Inf     1.179     1.457
##  Self-regulation         1.052 0.0687 Inf     0.918     1.187
##  Appreciation of beauty  0.746 0.0691 Inf     0.611     0.882
##  Gratitude               1.465 0.0691 Inf     1.330     1.601
##  Hope                    1.685 0.0694 Inf     1.549     1.821
##  Humor                   1.368 0.0678 Inf     1.235     1.501
##  Spirituality            0.795 0.0698 Inf     0.658     0.932
##  Machiavellianism       -1.839 0.0688 Inf    -1.974    -1.704
##  Narcissism             -0.839 0.0717 Inf    -0.979    -0.698
##  Psychopathy            -1.843 0.0675 Inf    -1.976    -1.711
## 
## Results are averaged over the levels of: Condition 
## Degrees-of-freedom method: asymptotic 
## Confidence level used: 0.95
```

```
ME2$`emmeans of Condition`
```

```
##  Condition        emmean     SE  df asymp.LCL asymp.UCL
##  no outcome       1.3931 0.0423 Inf    1.3101     1.476
##  positive outcome 1.6865 0.0423 Inf    1.6036     1.769
##  mixed outcome    0.6405 0.0423 Inf    0.5575     0.723
##  negative outcome 0.0481 0.0423 Inf   -0.0348     0.131
## 
## Results are averaged over the levels of: Strength 
## Degrees-of-freedom method: asymptotic 
## Confidence level used: 0.95
```

```
IA$`emmeans of Strength, Condition`
```

```
##  Strength               Condition         emmean    SE  df asymp.LCL asymp.UCL
##  Creativity             no outcome        1.3709 0.131 Inf    1.1143   1.62747
##  Curiosity              no outcome        1.5731 0.122 Inf    1.3346   1.81151
##  Judgment               no outcome        2.5280 0.114 Inf    2.3052   2.75081
##  Love of learning       no outcome        1.3591 0.126 Inf    1.1113   1.60687
##  Perspective            no outcome        1.2166 0.121 Inf    0.9796   1.45355
##  Bravery                no outcome        2.3543 0.122 Inf    2.1144   2.59417
##  Perseverance           no outcome        1.3006 0.115 Inf    1.0754   1.52573
##  Honesty                no outcome        2.4693 0.121 Inf    2.2324   2.70632
##  Zest                   no outcome        1.1489 0.129 Inf    0.8959   1.40183
##  Love                   no outcome        2.0597 0.125 Inf    1.8151   2.30419
##  Kindness               no outcome        2.6257 0.117 Inf    2.3955   2.85584
##  Social intelligence    no outcome        1.9710 0.115 Inf    1.7447   2.19740
##  Teamwork               no outcome        2.2142 0.117 Inf    1.9853   2.44304
##  Fairness               no outcome        2.5000 0.121 Inf    2.2630   2.73700
##  Leadership             no outcome        2.0833 0.117 Inf    1.8544   2.31217
##  Forgiveness            no outcome        1.0546 0.119 Inf    0.8218   1.28742
##  Humility               no outcome        2.1056 0.120 Inf    1.8700   2.34119
##  Prudence               no outcome        1.5478 0.124 Inf    1.3049   1.79080
##  Self-regulation        no outcome        1.3128 0.119 Inf    1.0786   1.54692
##  Appreciation of beauty no outcome        1.2592 0.120 Inf    1.0236   1.49476
##  Gratitude              no outcome        2.1561 0.120 Inf    1.9205   2.39170
##  Hope                   no outcome        2.3232 0.121 Inf    2.0862   2.56020
##  Humor                  no outcome        2.1029 0.117 Inf    1.8727   2.33306
##  Spirituality           no outcome        0.7975 0.122 Inf    0.5591   1.03594
##  Machiavellianism       no outcome       -2.3350 0.120 Inf   -2.5692  -2.10073
##  Narcissism             no outcome       -1.1274 0.126 Inf   -1.3736  -0.88116
##  Psychopathy            no outcome       -2.3592 0.117 Inf   -2.5882  -2.13029
##  Creativity             positive outcome  1.5935 0.131 Inf    1.3369   1.85012
##  Curiosity              positive outcome  1.8997 0.122 Inf    1.6613   2.13817
##  Judgment               positive outcome  2.1257 0.114 Inf    1.9029   2.34851
##  Love of learning       positive outcome  1.5439 0.126 Inf    1.2961   1.79166
##  Perspective            positive outcome  1.6870 0.121 Inf    1.4500   1.92395
##  Bravery                positive outcome  2.2495 0.122 Inf    2.0096   2.48944
##  Perseverance           positive outcome  1.7212 0.115 Inf    1.4960   1.94632
##  Honesty                positive outcome  2.3805 0.121 Inf    2.1435   2.61750
##  Zest                   positive outcome  1.5239 0.129 Inf    1.2709   1.77683
##  Love                   positive outcome  2.1266 0.125 Inf    1.8820   2.37109
##  Kindness               positive outcome  2.4621 0.117 Inf    2.2319   2.69226
##  Social intelligence    positive outcome  2.1258 0.115 Inf    1.8994   2.35216
##  Teamwork               positive outcome  2.2294 0.117 Inf    2.0005   2.45829
##  Fairness               positive outcome  2.5165 0.121 Inf    2.2795   2.75345
##  Leadership             positive outcome  2.1321 0.117 Inf    1.9032   2.36095
##  Forgiveness            positive outcome  1.3869 0.119 Inf    1.1541   1.61970
##  Humility               positive outcome  2.1706 0.120 Inf    1.9350   2.40612
##  Prudence               positive outcome  1.9159 0.124 Inf    1.6729   2.15886
##  Self-regulation        positive outcome  1.8063 0.119 Inf    1.5722   2.04051
##  Appreciation of beauty positive outcome  1.7819 0.120 Inf    1.5463   2.01748
##  Gratitude              positive outcome  2.2016 0.120 Inf    1.9660   2.43715
##  Hope                   positive outcome  2.3298 0.121 Inf    2.0928   2.56678
##  Humor                  positive outcome  2.2943 0.117 Inf    2.0641   2.52442
##  Spirituality           positive outcome  1.6408 0.122 Inf    1.4024   1.87928
##  Machiavellianism       positive outcome -0.9504 0.120 Inf   -1.1846  -0.71611
##  Narcissism             positive outcome  0.0405 0.126 Inf   -0.2057   0.28670
##  Psychopathy            positive outcome -1.3989 0.117 Inf   -1.6278  -1.16993
##  Creativity             mixed outcome     0.4060 0.131 Inf    0.1494   0.66262
##  Curiosity              mixed outcome     0.7031 0.122 Inf    0.4646   0.94151
##  Judgment               mixed outcome     1.1775 0.114 Inf    0.9547   1.40024
##  Love of learning       mixed outcome     0.4207 0.126 Inf    0.1729   0.66847
##  Perspective            mixed outcome     0.5028 0.121 Inf    0.2658   0.73974
##  Bravery                mixed outcome     1.7462 0.122 Inf    1.5063   1.98606
##  Perseverance           mixed outcome     0.0976 0.115 Inf   -0.1275   0.32279
##  Honesty                mixed outcome     1.8641 0.121 Inf    1.6271   2.10105
##  Zest                   mixed outcome     0.6451 0.129 Inf    0.3921   0.89804
##  Love                   mixed outcome     0.8519 0.125 Inf    0.6074   1.09644
##  Kindness               mixed outcome     1.2522 0.117 Inf    1.0221   1.48238
##  Social intelligence    mixed outcome     1.2032 0.115 Inf    0.9768   1.42954
##  Teamwork               mixed outcome     1.1806 0.117 Inf    0.9517   1.40951
##  Fairness               mixed outcome     1.3059 0.121 Inf    1.0690   1.54293
##  Leadership             mixed outcome     0.8150 0.117 Inf    0.5861   1.04387
##  Forgiveness            mixed outcome     0.5451 0.119 Inf    0.3123   0.77793
##  Humility               mixed outcome     0.6965 0.120 Inf    0.4610   0.93210
##  Prudence               mixed outcome     1.2006 0.124 Inf    0.9577   1.44358
##  Self-regulation        mixed outcome     0.8224 0.119 Inf    0.5882   1.05654
##  Appreciation of beauty mixed outcome     0.1683 0.120 Inf   -0.0673   0.40385
##  Gratitude              mixed outcome     0.8769 0.120 Inf    0.6413   1.11248
##  Hope                   mixed outcome     1.4186 0.121 Inf    1.1816   1.65560
##  Humor                  mixed outcome     0.9640 0.117 Inf    0.7338   1.19418
##  Spirituality           mixed outcome     0.6575 0.122 Inf    0.4191   0.89594
##  Machiavellianism       mixed outcome    -1.7452 0.120 Inf   -1.9795  -1.51098
##  Narcissism             mixed outcome    -0.9452 0.126 Inf   -1.1914  -0.69902
##  Psychopathy            mixed outcome    -1.5391 0.117 Inf   -1.7680  -1.31017
##  Creativity             negative outcome -0.3127 0.131 Inf   -0.5693  -0.05613
##  Curiosity              negative outcome  0.2164 0.122 Inf   -0.0220   0.45484
##  Judgment               negative outcome  0.6085 0.114 Inf    0.3857   0.83127
##  Love of learning       negative outcome -0.1409 0.126 Inf   -0.3887   0.10687
##  Perspective            negative outcome  0.1212 0.121 Inf   -0.1158   0.35816
##  Bravery                negative outcome  1.2664 0.122 Inf    1.0265   1.50633
##  Perseverance           negative outcome -0.5818 0.115 Inf   -0.8069  -0.35662
##  Honesty                negative outcome  1.2621 0.121 Inf    1.0251   1.49908
##  Zest                   negative outcome -0.1731 0.129 Inf   -0.4261   0.07986
##  Love                   negative outcome -0.3101 0.125 Inf   -0.5546  -0.06553
##  Kindness               negative outcome  1.1072 0.117 Inf    0.8770   1.33732
##  Social intelligence    negative outcome  0.6615 0.115 Inf    0.4351   0.88788
##  Teamwork               negative outcome  0.1928 0.117 Inf   -0.0361   0.42170
##  Fairness               negative outcome  0.5954 0.121 Inf    0.3584   0.83240
##  Leadership             negative outcome  0.2083 0.117 Inf   -0.0206   0.43717
##  Forgiveness            negative outcome -0.2239 0.119 Inf   -0.4567   0.00894
##  Humility               negative outcome  0.3848 0.120 Inf    0.1493   0.62041
##  Prudence               negative outcome  0.6069 0.124 Inf    0.3639   0.84983
##  Self-regulation        negative outcome  0.2679 0.119 Inf    0.0337   0.50205
##  Appreciation of beauty negative outcome -0.2246 0.120 Inf   -0.4602   0.01099
##  Gratitude              negative outcome  0.6269 0.120 Inf    0.3913   0.86248
##  Hope                   negative outcome  0.6686 0.121 Inf    0.4316   0.90560
##  Humor                  negative outcome  0.1122 0.117 Inf   -0.1180   0.34232
##  Spirituality           negative outcome  0.0842 0.122 Inf   -0.1543   0.32261
##  Machiavellianism       negative outcome -2.3254 0.120 Inf   -2.5596  -2.09111
##  Narcissism             negative outcome -1.3238 0.126 Inf   -1.5700  -1.07759
##  Psychopathy            negative outcome -2.0757 0.117 Inf   -2.3046  -1.84676
## 
## Degrees-of-freedom method: asymptotic 
## Confidence level used: 0.95
```
